# Roles of Microglial and Monocyte Chemokines and Their Receptors in Regulating Alzheimer's Disease-Associated Amyloid-β and Tau Pathologies

**DOI:** 10.3389/fneur.2018.00549

**Published:** 2018-08-14

**Authors:** Joana R. Guedes, Taotao Lao, Ana L. Cardoso, Joseph El Khoury

**Affiliations:** ^1^Doctoral Programme in Experimental Biology and Biomedicine, CNC - Center for Neuroscience and Cell Biology, University of Coimbra, Coimbra, Portugal; ^2^Institute for Interdisciplinary Research, University of Coimbra, Coimbra, Portugal; ^3^CNC - Center for Neuroscience and Cell Biology, University of Coimbra, Coimbra, Portugal; ^4^Center for Immunology and Inflammatory Diseases, Massachusetts General Hospital, Harvard Medical School, Charlestown, MA, United States; ^5^Division of Infectious Diseases, Massachusetts General Hospital, Harvard Medical School, Charlestown, MA, United States

**Keywords:** Alzheimer's disease, amyloid-β peptide, protein tau, microglia, monocytes, chemokine receptors, chemokines, neuroinflammation

## Abstract

Chemokines and their receptors have been shown to affect amyloid-β (Aβ) and tau pathologies in mouse models of Alzheimer's disease (AD) by regulating microglia and monocyte-associated neuroinflammation, microglial movement and monocyte recruitment into the brain. These cells in turn can promote and mediate Aβ phagocytosis and degradation and tau phosphorylation. In this review we discuss published work in this field in mouse models of AD and review what is known about the contributions of microglial and monocyte chemokines and their receptors to amyloid and tau pathologies. We focus on the roles of the chemokine/chemokine receptor pairs CCL2/CCR2, CX3CL1/CX3CR1, CCL5/CCR5, CXCL10/CXCR3 and CXCL1/CXCR2, highlighting important knowledge gaps in this field. A full understanding of the functions of chemokines and their receptors in AD may guide the development of novel immunotherapies for this devastating disease.

## Introduction

Neuroinflammation is an important contributor to Alzheimer's disease (AD) pathogenesis and progression (1, 2). Indeed, several inflammatory mediators such as tumor necrosis factor (TNF) and interleukin-1 (IL-1) are elevated in the brains of AD patients and mouse models of AD (3, 4). In addition, several variants in immune genes such as TREM2, CD33, and CR1 that regulate the inflammatory response have been identified by Genome-Wide Association Studies (GWAS) as genetic risk factors for AD (5–9). Microglia and recruited peripheral blood monocytes are the principal innate immune cells involved in the pathogenesis of AD and extensive evidence indicate that their functions are in part regulated by chemokines and their receptors (1).

## Beta amyloid deposition and Tau hyperphosphorylation are hallmarks of AD

Clinically, AD is associated with dementia and progressive cognitive decline. Pathologically, AD is characterized by the presence of extracellular senile plaques and intraneuronal neurofibrillary tangles (NFTs). These protein deposits contain aggregates of the amyloid-β (Aβ) peptide and hyperphosphorylated microtubule-associated protein tau (p-τ), respectively. The processes of Aβ production and tau hyperphosphorylation involve different pathways. Aβ accumulation is the result of tandem cleavage of the amyloid precursor protein (APP) by secretases, whereas tau hyperphosphorylation results from the activity of several kinases. However, increasing evidence indicate that these abnormal protein deposits influence each other and have an additive effect on disease progression and that Aβ deposition appears to regulate tau pathology (10, 11). Indeed, in experimental animals, Aβ injection in the brain of P301L mice, a model for tau pathology, increases formation of NFTs (12). Furthermore, breeding mice expressing 5 early onset familial AD mutations (5XFAD) with mice expressing the tau P301S mutation (PS19) results in a ~10-fold aggravated tauopathy (13). In contrast, APP-KO mice subjected to subtoxic doses of soluble oligomeric forms of tau protein do not exhibit tau-induced defects in spatial/associative memory and its electrophysiological surrogate long-term potentiation (LTP), suggesting that tau pathology is dependent on APP expression (14). Aβ accumulation appears to promote tau hyperphosphorylation via activation of glycogen synthase kinase-3β (GSK-3β) (15).

The effects of Aβ deposition on tau pathology and neurodegeneration may not be necessarily due only to fibrillar Aβ deposits in senile plaques. In fact, cognitive deficits start before these plaques are visible and it has been proposed that the spectrum of Aβ species, including intraneuronal Aβ, soluble Aβ and Aβ oligomers also contribute to synaptic disruption and tau hyperphosphorylation. Regardless of the exact species of Aβ involved in this process, the co-localization of Aβ and p-τ in synaptic sites (16) suggest that the deposition of these protein aggregates and their contribution to neuronal loss is affected by their interactions. In this regard, strategies for treatment of AD should consider both Aβ and p-τ aggregates and how they influence each other (17).

## The mononuclear phagocyte system in alzheimer's disease

There is strong supporting evidence that the inflammatory response in AD is in part driven by the interaction of Aβ with mononuclear phagocytes, including microglia and recruited peripheral blood monocytes (1, 18, 19). Multiple forms of Aβ accumulate in the AD brain before the development of visible senile plaques and formation of NFT. Since these forms of Aβ can interact with microglia and/or monocytes and lead to an inflammatory response, several groups have suggested that neuroinflammation is an early event in Aβ and tau pathologies (20–22), preceding the accumulation of larger visible protein deposits. The mononuclear phagocyte system has been extensively studied in the context of neuroinflammation in AD. However, the contribution of different cells types of this system, such as microglia and monocytes to AD pathogenesis is only beginning to be understood.

Microglia are the principal resident sentinels in the brain and can rapidly sense changes in their environment such as Aβ deposition, using a set of genes termed the sensome (23). Subsequent to sensing Aβ, the interaction of microglia with Aβ is a double-edged-sword. On one hand, microglia can phagocyte and clear Aβ deposits thereby limiting the progression of AD pathology (24, 25). In support of this pathway, reduced expression of Aβ-binding receptors in microglia from aged mice is associated with reduced Aβ phagocytosis and clearance and increased Aβ accumulation and disease progression (4). In contrast, the continuous interaction of microglia with Aβ induces the activation of the inflammasome pathway and produce several inflammatory mediators and neurotoxins, thereby contributing to the progression of AD in several ways (2, 19, 26–30). It is possible that persistent Aβ-induced microglial activation could also contribute to Aβ deposition, since continuous production of neurotoxic factors by microglia can induce neuronal apoptosis and consequent release of intraneuronal Aβ into the extracellular space. In addition, inflammatory cytokines as IL-1β, INF-γ, and TNF-α have been shown to upregulate β-secretase expression thereby increasing Aβ production (31). Such inflammatory cytokines also affect LTP in the hippocampus (32), suggesting that they can influence synaptic plasticity, which is essential for memory formation. In support of this, during microglia activation, their branches disappear, giving place to an amoeboid morphology which may limit their ability to refine and sustain synapses.

In contrast to what we know about microglia-Aβ interactions, less is known about microglia and NFTs. Microglia appear to promote tau propagation and contribute to the spreading of tau pathology in the brain (33, 34). Microglial activation also induces tau phosphorylation and aggregation (35). Some anti-inflammatory drugs have been shown to reduce p-τ in P301S and 3xTg AD mice (22, 36), but whether the mechanism is microglia-dependent remains to be determined. Published reports also suggest that injured neurons exhibiting tau hyperphosphorylation modulate microglia-mediated neuroinflammation (37). While these studies are compelling, they do not provide evidence of direct activation of microglia by p-τ. However, they suggest that neuroinflammation is closely linked to tau pathology.

In addition to studies on microglia function in AD, monocytes can infiltrate the AD brain and, although their role in AD progression is unclear at this time, it has been proposed that they have both detrimental and beneficial effects depending on the stage of disease development (38–40). Monocytes infiltrate the AD brain and clear perivascular Aβ (39, 41, 42). In support of this, stimulating monocyte infiltration by peripheral challenging with macrophage colony-stimulating factor (M-CSF) and lipopolysaccharide (LPS) or blocking immunosuppressive transforming growth factor beta (TFG-β) can attenuate AD pathology (43). While these studies suggest a protective role for monocytes in AD, monocytes, similar to microglia, can become activated and promote neuroinflammation and neurotoxicity (44).

In contrast to the studies supporting a role of monocytes in AD, a recent report using parabiosis experiments and staining for CD11b and CD45 suggests that monocytes do not infiltrate the brain in APP/PS1 and 5XFAD mouse models of amyloidosis (45). Interestingly, the same group also showed the presence of monocytes in the brain of 5XFAD mice when single cell RNAseq was performed on myeloid cells and more cell specific markers were analyzed (46). Additional mouse and human studies using single cell RNASeq, flow cytometry with multiple microglia and monocyte markers and *in situ* hybridization will help clarify this apparent discrepancy.

## Chemokines are essential for the accumulation of mononuclear phagocytes in the alzheimer's disease brain

Chemokines are chemotactic cytokines which mediate immune cells migration to sites of inflammation. Initially designated with specific protein names, chemokines are now classified based on the number of amino acids between two cysteine residues: α-chemokines, with the first cysteine residues separated by one amino acid (CXC); β-chemokines, with adjacent cysteine residues (CC); lymphotactin, with only two cysteines, and fractalkine (CX3CL1) in which the first two cysteine residues are separated by three amino acids (47, 48). These small chemoattractant proteins bind to chemokine receptors classified in the same manner. Different chemokine receptors are expressed on different immune cells. Monocytes, for example, express CCR1, CCR2, CCR5, CCR8, CXCR4, and some CX3CR1 (49). In contrast, microglia express very high levels of CX3CR1 and CCR5 and, to a lesser extent CXCR4, CXCR3, and CXCR2 (23).

Since chemokines mediate the infiltration of peripheral monocytes into the inflamed central nervous system (50), we proposed that the same occurs in AD (47, 48). The association between chemokines and AD is supported by studies showing that CCL2 and CCL5 expression are increased in the AD brain (51, 52). *In vitro*, microglia express CCL2 when incubated with Aβ (19) and neurons from AD brains have been shown to upregulate CCL5 expression (53). Moreover, CCL5 is essential to Aβ induced-microglia chemotaxis (54, 55). Similarly, the CX3CR1 ligand CX3CL1 is highly expressed on neurons. These studies suggest that chemokines are important in AD and possibly mediate the infiltration of peripheral monocytes into the AD brain and/or the accumulation of microglia at sites of Aβ deposition. Additional insights into the roles of chemokines, their receptors and mononuclear phagocytes in AD progression come from animal models where the function of chemokine receptors have been challenged.

## CCL2/CCR2 axis

When combined with Aβ and p-τ levels, CCL2 expression in the brain and cerebrospinal fluid (CSF) is a reliable predictor of AD severity (51, 56). In support of these observations, the CCL2 receptor, CCR2, was the first chemokine receptor shown to be associated with AD. We found that, in Tg2576 AD mice, CCR2 deficiency accelerates early disease progression by impairing the accumulation of mononuclear phagocytes (39). APP-*CCR2*^−/−^ mice exhibited higher Aβ levels and reduced CD11b^+^ cell recruitment into the brain. Importantly, these mice showed higher mortality in a *CCR2* gene dosage-dependent manner. Subsequent studies showed that the decrease in perivascular Aβ observed in our study was due to the lack of blood monocytes accumulation at these sites and possibly their infiltration in the brain parenchyma (39). These findings were corroborated by the finding that CCR2 deficiency worsened memory deficits and increased soluble Aβ in APP/PS1 mice (57). This study also showed that lack of CCR2 stimulated the expression of TGF-β receptors and CX3CR1 in plaque-associated microglia, implicating another chemokine receptor in AD pathology. The progressive cognitive decline in these mice was associated with a decrease in the numbers of CX3CR1^low^Ly6-C^high^CCR2^+^Gr1^+^ circulating inflammatory monocytes (58) and restoring CCR2 expression in bone marrow cells reestablished memory capacities and decreased soluble Aβ accumulation (58). Altogether, these reports show that CCR2^+^ monocytes can be protective in AD.

**Figure 1:**
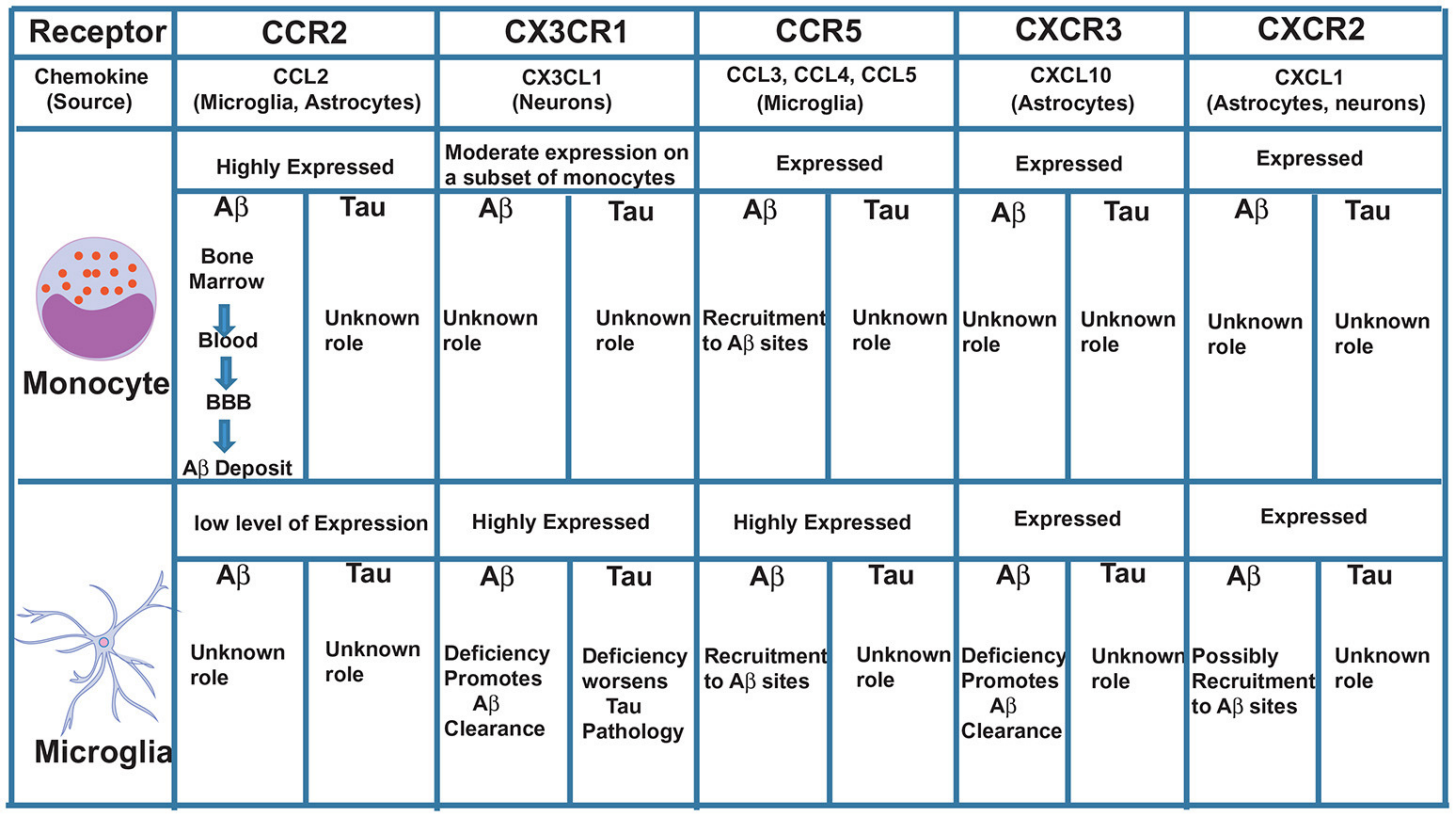
Major chemokine receptors expressed on monocytes and microglia and their possible roles in Aβ and Tau pathologies.

We propose that CCR2 promotes the recruitment of monocytes initially from the bone marrow into the blood, then from the blood across the blood-brain barrier (BBB), to the perivascular space, then from the perivascular space to sites of Aβ deposition in the parenchyma where these cells can potentially clear Aβ by phagocytosis (Figure 1). In support of these findings, we recently showed that the CCL2/CCR2 axis was impaired in blood-derived monocytes from AD patients, causing a deficit in cell migration (59).

## CX3CL1/CX3CR1 axis

Another chemokine receptor thoroughly studied in AD is CX3CR1. This receptor is highly expressed in microglia and CX3CR1-GFP *knock-in* mice (where GFP replaced one CX3CR1 allele) have been used to specifically study, *in vivo*, the role of microglia in AD and other brain diseases. In physiological conditions, disruption of CX3CR1 function affects cognitive functions in a IL-1β-dependent manner (60) and exacerbates LPS-induced inflammation (61), suggesting that CX3CR1 maintains microglial homeostasis, being essential for their function in synaptic support and limiting their activation. In contrast to its clear role in maintaining microglial homeostatic functions under physiological conditions, dysregulation of the CX3CL1/CX3CR1 axis in AD mouse models can have both neuroprotective and neurotoxic effects depending on the mouse model used. CX3CR1 deficiency in three different AD mouse models—APP/PS1, R1.40 and CRND8—reduced amyloid deposits and enhanced Aβ phagocytic ability by microglia (62, 63). These effects were associated with decreased microglia activation and TNF levels and increased IL-1β levels. Similarly, following injection of Aβ_1−40_ fibrils in the hippocampus, downregulation of CX3CR1 with siRNAs also suppressed microglial activation, increasing synaptic strength and cognitive functions (64). On the other hand, CX3CR1 deficiency in hAPP mice worsened behavioral deficits associated with cytokine production independent of plaques deposition (65). Although these mice do not express tau mutations, the effect of CX3CR1 disruption was associated with enhanced tau pathology. Moreover, hTau mice (which exhibit p-τ) lacking CX3CR1 exhibited enhanced tau phosphorylation and aggregation associated with microglial activation, as well as behavioral impairments (35, 66). Analyzing these studies, it is interesting to observe that the neuroprotective role of CX3CR1 is always associated with tau and not Aβ. In support of these findings, overexpression of soluble CX3CL1 in mice with Tau but not Aβ pathology led to substantial improvements (67). Interestingly, CX3CR1 deletion in 3xTg mice, which have both Aβ and tau pathologies, prevented neuronal loss, suggesting that the effect of CX3CR1 deficiency on Aβ pathology may be more dominant than its effect on tau pathology (68). It is possible, however, that CX3CR1 is involved in the killing of neurons with intracellular tau deposits and the subsequent release of tau (68) (Figure 1). Additional studies with AD models that exhibit both Aβ and tau pathologies are needed to definitively clarify the role of CX3CR1 in AD.

## CCL5/CCR5 axis

CCR5^+^ reactive microglia are found associated with Aβ deposits in AD patients (69). CCR5^−/−^ mice have higher levels of Aβ, C99 [a product that results from APP cleavage by β-secretase 1 (BACE1)] and BACE1 itself compared to normal mice (70). These levels were associated with astrocyte activation and CCR2 overexpression leading to cognitive impairments. Also, following injection of Aβ_1−40_ into the lateral ventricle of CCR5^−/−^ mice, a reduced activation of microglia and astrocytes was observed compared to wild-type mice (71). Interestingly, CCR5 appears to be essential for the transendothelial migration of T cells across the BBB in the hippocampus of rats injected with Aβ (72), suggesting that CCR5 may be essential for the infiltration of non-phagocytic peripheral immune cells to the AD brain. Since CCR5 is also expressed on a subset of monocytes and on microglia, it is possible that it plays an additive role to CCR2 in mediating monocyte movement across the BBB, as well as in the movement of microglia toward sites of Aβ deposition in the parenchyma (Figure 1).

In addition to CCL5, we have shown that microglia and macrophages stimulated with Aβ, *in vitro*, show increased mRNA levels for the chemokines CCL3 and CCL4 also known as MIP1α and MIP1β respectively (19). These two chemokines are also upregulated in adult human microglia isolated from post-mortem brains and stimulated with Aβ (73) and in plaque-associated microglia in AD patients (74). The exact roles of these chemokines in AD is not clear. However, both chemokines are ligands for CCR5, suggesting possible complementary roles to CCL5 in the accumulation of T cells, monocytes or microglia in the AD brain. To date, there are no published reports describing the effects of CCL3, CCL4, or CCL5 deficiency on Aβ deposition or tau pathology in a transgenic Aβ or tau model. Such studies will help clarify the role of CCR5 ligands in AD development and progression, an important current knowledge gap in the field.

## CXCL10/CXCR3 axis

CXCL10 levels in CSF are significantly increased in patients with amnestic mild cognitive impairment (MCI) and patients with mild AD, but not in patients with severe AD (75). Unlike its receptor CXCR3, which is constitutively expressed on microglia and neurons, CXCL10 is expressed in a subpopulation of astrocytes in the normal brain and is markedly elevated in astrocytes in AD (76, 77). Peripheral injection of LPS strongly induces CXCL10 the brain of rats, as well as in cultured astrocytes and microglia (78), indicating its involvement in the response to inflammatory stimuli. Although CXCL10 was found to co-localize with Aβ plaques in APP mice (79), its function in regulating Aβ pathology remains unclear. A recent study using CXCR3-deficient AD mice showed that deletion of CXCR3 significantly reduced plaque burden and Aβ levels in APP/PS1 mice with morphological evidence for microglial activation, but reduced plaque association. This study suggests a possible role in the recruitment of microglia to Aβ deposits (80). CXCR3 deficiency also increased microglial uptake of Aβ, reduced concentrations of proinflammatory cytokines and attenuated behavioral deficits in APP/PS1 mice (80), suggesting an important role for CXCL10/CXCR3 signaling in mediating Aβ-induced pathology in mouse models. The role of CX3CR1 in tau pathology is not clear and needs to be investigated (Figure 1).

## CXCL1/CXCR2 axis

CXCR2 is expressed at low levels on microglia (23). CXCR2 deficiency results in reduction of Aβ with concurrent increases of γ-secretase substrates in the APP/PS1 mice (81). Whether microglia play a direct role in this process is not clear and requires a more detailed analysis of CXCR2- deficient APP/PS1 mice. Insights into a possible microglial role on the effect of CXCR2 depletion came from studies performed in rats injected with Aβ. Administration of a competitive CXCR2 antagonist to Aβ-injected rats significantly reduced expression of CXCR2 and microgliosis (82). Similar studies in mouse models of AD need to be done to further validate these findings.

## Chemokines receptors as targets for AD therapy

Based on the above discussion, it may be possible, in the future, to consider chemokine receptors as potential targets for the treatment of AD. One could envision upregulating CCR2 expression to increase influx of monocytes into the AD brain, therefore leading to increased removal of Aβ deposits and reduced Aβ burden. However, it will be difficult to devise such a strategy for CX3CR1 and CCR5, until the effects of deleting these receptors on both Aβ- and tau-associated pathologies are defined in animal models that exhibit both features. Importantly, one must consider that these receptors can display different functions in different stages of AD progression.

## Conclusion

The work summarized in this review suggests that chemokines and their receptors are important for AD pathogenesis and in the development of the two main pathological hallmarks of AD—Aβ deposition and tau hyperphosphorylation. These receptors are associated with innate and adaptive responses regulating microglia and peripheral immune cells activation, and are essential for the infiltration of immune cells to the AD brain and movement of recruited monocytes and microglia toward sites of Aβ deposition. These studies clearly point to the importance of the neuroinflammatory component of AD as an active process which contributes to AD development and suggest that the presence of immune cells in the AD brain is tightly regulated during different stages of AD progression, constituting a double-edged sword that may lead to neuroprotective or neurotoxic outcomes. However, in spite of the significant amount of knowledge gained so far, there are important knowledge gaps that limit our understanding of the roles of chemokines and their receptors in AD. Additional studies to explore how these receptors and their ligands influence Aβ deposition, tau hyperphosphorylation, microglia and monocyte accumulation, the overall inflammatory response and neuronal degeneration are needed. A full understanding of the roles of chemokines and their receptors in AD may guide the development of multiple novel immunotherapies targeted to various stages of the disease.

## Author contributions

JRG and JE wrote the initial version of the manuscript, then all authors contributed to writing and editing the submitted manuscript.

### Conflict of interest statement

The authors declare that the research was conducted in the absence of any commercial or financial relationships that could be construed as a potential conflict of interest.
